# Combination Therapy with Disulfiram, Copper, and Doxorubicin for Osteosarcoma: *In Vitro* Support for a Novel Drug Repurposing Strategy

**DOI:** 10.1155/2019/1320201

**Published:** 2019-07-11

**Authors:** Jonathan B. Mandell, Feiqi Lu, Matthew Fisch, Jan H. Beumer, Jianxia Guo, Rebecca J. Watters, Kurt R. Weiss

**Affiliations:** ^1^Musculoskeletal Oncology Laboratory, Department of Orthopaedic Surgery, University of Pittsburgh, Pittsburgh, PA, USA; ^2^Department of Infectious Diseases and Microbiology, University of Pittsburgh, Pittsburgh, PA, USA; ^3^School of Medicine, Tsinghua University, Beijing, China; ^4^Department of Pharmaceutical Sciences, University of Pittsburgh, Pittsburgh, PA, USA; ^5^UPMC Hillman Cancer Center, Pittsburgh, PA, USA; ^6^Department of Pharmacology and Chemical Biology, University of Pittsburgh, Pittsburgh, PA, USA; ^7^Departments of Anatomic Pathology and General Surgical Oncology, University of Pittsburgh, Pittsburgh, PA, USA

## Abstract

Although many cancer cells have significantly higher copper concentrations compared with normal cells and tissues, the role of copper in cancer biology and metastatic disease remains poorly understood. Here, we study the importance of copper in osteosarcoma, which frequently metastasizes to the lungs and is often chemoresistant. K12 and K7M2 are murine OS cells with differing metastatic phenotypes: K7M2 is highly metastatic, whereas K12 is much less so. Intracellular copper levels were determined using atomic absorption. Copper transporters were quantified by qPCR. Cytotoxicity of doxorubicin, disulfiram, and copper(II) chloride was assessed with a cell viability fluorescence stain. Additionally, K7M2 viable cell counts were determined by trypan blue exclusion staining after 72 hours of treatment. Copper levels were found to be significantly higher in K12 OS cells than in K7M2 cells. qPCR showed that K12 cells upregulate the copper influx pump CTR1 and downregulate the copper efflux pump ATP7A compared to K7M2 OS cells. Combination treatment of copper chloride (50 nM) with disulfiram (80 nM) was only cytotoxic to K12 cells. Triple treatment with doxorubicin, disulfiram, and copper displayed potent and durable cytotoxicity of highly metastatic K7M2 cells. We demonstrate here that murine OS cell lines differing in metastatic potential also vary in endogenous copper levels and regulation. Additionally, these differences in copper regulation may contribute to selective cytotoxicity of K12 cells by extremely low doses of copper-potentiated disulfiram. The combination of doxorubicin, disulfiram, and copper should be explored as a therapeutic strategy against OS metastases.

## 1. Introduction

Osteosarcoma (OS) is the most common primary malignancy of the bone and mainly afflicts children and teens. Virtually, all metastatic disease is to the lungs, which ultimately causes patient mortality. Before the chemotherapeutic era, 5-year survival with surgery alone was only 10–20%. Modern treatment paradigms employ neoadjuvant (preoperative) chemotherapy, surgery, and adjuvant (postoperative) chemotherapy. The combination of cytotoxic chemotherapy and surgical ablation has improved 5-year survival to approximately 65% in most large series. Although several chemotherapeutic agents are used to treat OS, doxorubicin (Dox) has functioned as the backbone of OS chemotherapy for decades. Dox is considered to be an indispensable component for worldwide treatment of OS [1–12].

The addition of Dox to the treatment OS has dramatically improved outcomes, but enthusiasm is limited by several realities. Most importantly, the prognoses of children with OS have not improved in over three decades despite multiple clinical trials [13–17]. Children who present with pulmonary metastases at the time of diagnosis, or develop them during the course of their treatment, have especially poor prognoses of 15–30% 5-year survival [6]. Additionally, children who survive OS do so at great cost. Dox is extraordinarily cardiotoxic, causing dose-dependent cardiomyopathy that leads to congestive heart failure and death. Two recent reviews of long-term pediatric sarcoma survivors found incidences of severe cardiotoxicity of 26% and 28% [18, 19]. Therefore, the two greatest obstacles that prevent improvement of the prognoses of OS patients are: (1) the absence of treatments that specifically target OS metastatic biology, and (2) the deleterious long-term consequences of Dox treatment for OS survivors. The efficacy of conventional cytotoxic chemotherapy regimens in OS have likely reached their zenith, necessitating the application of more biologically intelligent approaches.

K7M2 and K12 are murine OS cell lines that are both derived from the same parental tumor in Balb/cJ mouse. K7M2 is aggressively metastatic to the lung, whereas K12 displays a weakly metastatic phenotype. As these cell lines are clonally related but display dramatically different metastatic abilities, they are a simple yet powerful tool to study OS metastatic biology [20, 21]. Previous work from our group utilized K7M2 and K12 to identify and investigate factors that confer metastatic potential to OS cells [22–25]. K7M2 cells express and produce significantly greater amounts of the cancer stem cell marker aldehyde dehydrogenase (ALDH) compared with K12 cells, suggesting that ALDH is a metastasis-associated factor in OS [23, 24, 26]. ALDH enables OS cells to withstand oxidative stress, and OS cells with high ALDH expression are more motile and invasive than OS cells with lower ALDH expression. These findings led us to investigate the ability of the ALDH inhibitor, disulfiram (Dis), to alter the metastatic phenotypes of OS cells. We observed that Dis renders OS cells more susceptible to oxidative stress, alters OS cell morphology, and diminishes their viability and invasiveness. We also demonstrated dose-dependent cytotoxicity of Dis in the cultured cells of bone sarcoma patients from our clinic [27].

Copper (Cu) is an essential micronutrient for physiologic redox reactions and also important to the oncogenic processes of invasion and metastasis [28, 29]. Many neoplasia conditions display higher levels of endogenous intratumoral Cu compared with normal cells and tissues [30, 31]. Patients with more aggressive and chemotherapy-resistant diseases have demonstrated elevated serum Cu levels [30, 31]. Several basic and clinical studies have suggested that Dis's antitumor activity is enhanced by the addition of Cu(II) compounds [28, 32–37]. However, evaluation of the differences in Cu metabolism between low grade vs. highly metastatic tumors and how this directly relates to the success of Dis + Cu(II) treatment have not been explored.

In this report, we investigated differences in Cu metabolism and Dis + CuCl_2_ treatment between K12 and K7M2 cells. Intracellular Cu levels of K7M2 and K12 cells with and without treatments were also measured. We also determined expression levels of genes related to both Cu transport and chemotherapy resistance. Finally, we tested the ability of CuCl_2_ to alter the potency of Dis treatment in murine OS cells, as well as the potential of Dis + CuCl_2_ to reduce the cytotoxic dose of Dox. We hypothesized that OS cells with differing metastatic potentials possess varied endogenous Cu levels and altered gene expression patterns relating to Cu metabolism and drug resistance. We suspected that Cu would alter susceptibility of OS cells to Dis-mediated toxicity, and that the combination of Dis + CuCl_2_ might permit cytotoxicity with lower Dox doses.

## 2. Materials and Methods

### 2.1. Cell Lines and Cell Culturing

The highly metastatic OS K7M2 cell line was purchased from ATCC, and K12 cells were generously provided by Dr. Chand Khanna in the Pediatric Oncology Branch of the National Cancer Institute. Both cell lines were sent to IDEXX BioResearch labs to test for the presence of pathogens and mycoplasma, interspecies contamination, and STR profiling to definitively prove a positive identity of the clonally related OS cell lines. Both cell lines were grown and maintained using identical culture media and conditions. Cells were grown in T75 flasks (Corning) with culture media of DMEM (Corning) supplemented with 10% FBS (Gibco) and 1% penicillin/streptomycin Ab solution (Gibco) at 37°C in a humidified incubator.

### 2.2. Atomic Absorption (AA) Spectrophotometric Cu Analysis

At ∼80% confluency, both cell lines were washed with sterile Dulbecco's phosphate buffered saline (dPBS) and treated with TrypLE (Gibco) for 8 minutes at 37°C. Cells were given FBS (Gibco) containing culture media and centrifuged at 1500 rpm for 6 minutes. Cell pellets were washed with ice cold, sterile dPBS (Gibco), and cell counts were obtained using an automated cell counter (Bio-Rad). Cells were washed again with ice cold dPBS, centrifuged, and dry cell pellets were stored at −80°C. Additionally, K7M2 and K12 were treated over 24 hours with Dis (3 *μ*M), CuCl_2_ (1 *μ*M), and Dis + CuCl_2_ (0.1 and 0.25 *μ*M) then processed in the identical manner as untreated cells.

Copper concentrations were determined using a Perkin Elmer AAnalyst 600 atomic absorption spectrophotometer adjusted to detect Cu (324.8 nm). The AAS was operated as a flameless furnace system. Calibration samples were prepared by diluting the copper stock solution with 1% HNO_3_ to generate concentrations of 20, 50, 100, 200, 500, and 1000 ng/mL. Quality control samples were prepared at 60, 400, and 800 ng/mL.

Modifier solution consisted of 0.1% Pd and 0.06% Mg(NO_3_)_2_ in water, which was mixed 1 : 1 (v/v) with sample before injecting 10 *μ*L. Temperature programming was as per instruction manual. Cell pellets (minimum 2·10^5^ cells) were lysed with 35 *μ*L 0.25% triton. An aliquot of 5 *μ*L was taken for determination of protein using the Bio-Rad Protein assay following the manufacturer's instructions with bovine serum albumin as the standard. A further 30 *μ*L of cell lysate was incubated with 30 *μ*L of 63% HNO_3_ overnight at 60°C. Next, the sample was diluted with 40 *μ*l of 0.2% HNO_3_ and next diluted with modifier. Any other dilutions of samples (prior to addition of modifier) were done with 0.2% HNO_3_. The final concentration of Cu in media samples was calculated as total *μ*g/ml. Intracellular Cu concentration was calculated as: (final sample concentration of Cu (ng/mL))/(total sample protein concentration (mg/ml)) and recorded as ng·Cu/mg Protein.

### 2.3. Quantitative PCR

mRNA was collected from K12 and K7M2 using RNeasy Kit (Qiagen), and cDNA was obtained using Reverse Transcriptase Kit (Applied Biosystems). qPCR was performed using SYBR Green Supermix (BioRad). NoNo, Rps17, and Rps18 reference genes were selected from our previous work [38] for the most stable expression across multiple murine cell and tissue types. Delta CT values were derived from the geometric mean of the three selected housekeeper genes. Differential gene expression between K12 and K7M2 was determined for Cu influx pump (CTR1) and Cu efflux pump (ATP7A), aldehyde dehydrogenase (ALDH), and P-glycoprotein (ABCB1).

### 2.4. Cytotoxicity Assays

K12 and K7M2 were plated in black-walled 96-well plates (5,000 and 10,000 cells, respectively) and cultured for 24 hours. Media was replaced with culture media with fold dilutions of Dox, CuCl_2_, and Dis (1 nM–10 *μ*M). Additionally, Dis fold dilutions were tested in combination with fixed concentrations of CuCl_2_ (50, 200, and 800 nM). 24 hours after drug addition, cells were stained with a mammalian LIVE/DEAD cell viability kit (Invitrogen). Microscopy of cells was performed with a Nikon ECLIPSE TE2000 U using Northern Eclipse software. Live signal (%) reduction from the untreated controls was determined using FIJI image mean fluorescence intensity.

### 2.5. K7M2 Cytotoxicity and Recovery Assays

K7M2 cells were plated (0.1 × 10^6^) in 6-well plates and cultured for 24 hours. Cells were treated over 72 hours with CuCl_2_ (0.12–2 *μ*M), Dis (0.5–4 *μ*M), Dox (0.02–1.2, 10 *μ*M), and Dis (0.12–2 *μ*M) + CuCl_2_ (0.12–0.25 *μ*M). Cells were washed with dPBS, treated with 100 *μ*L TrypLE Express, and resuspended in culture media. Viable cell counts were obtained using a Trypan Blue exclusion assay and a hemocytometer. Treated K7M2 were recultured with fresh media without drugs in 6-well plates and monitored for robust recovery of cells. Additionally, K7M2 were treated with sublethal doses of Dox (100 nM), Dis (100 nM), and CuCl_2_ (250 nM) alone, in combination treatments, and triple combination treatments.

### 2.6. Statistics

All statistical methods were performed using Prism 7.0 (GraphPad, La Jolla CA). Multiple groups were compared using an ordinary one-way ANOVA. Two groups were compared using an unpaired *t*-test. In all cases, *p* < 0.05, *p* < 0.005, *p* < 0.0005, and *p* < 0.0001 were considered significant, as indicated in each figure.

## 3. Results

### 3.1. Less Metastatic K12 OS Cells Display Higher Endogenous and Therapeutic Levels of Intracellular Cu Compared to Highly Metastatic K7M2

**Figure 1:**
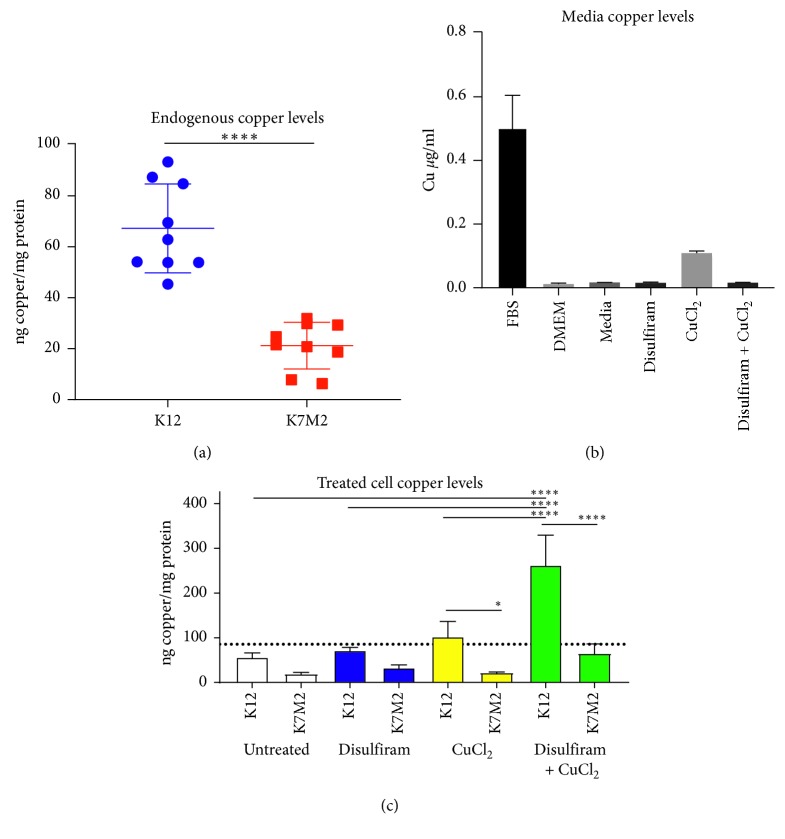
Murine OS cell line K12 displays higher levels of intracellular Cu compared to K7M2. Clonally related OS cell lines (K12 and K7M2) were cultured under identical conditions and then collected for protein quantification and intracellular Cu analysis using atomic absorption (AA) spectrophotometry. (a) Low metastatic K12 cells displayed significantly higher intracellular Cu levels than K7M2 (*n*=6). (b) AA analysis of media components and media containing Dis (3 *μ*M), CuCl_2_ (1 *μ*M), and Dis + CuCl_2_ (0.1 and 0.25 *μ*M, respectively) was performed (*n*=3). (c) Both K12 and K7M2 cells were cultured or media treated with Dis, CuCl_2_, or Dis + CuCl_2_ at doses previously AA tested (*n*=3). Copper analysis was performed using three independently cultured and processed samples. ^*∗*^*p* < 0.05; ^*∗∗∗∗*^*p* < 0.0001.

We first assessed differences in endogenous intracellular Cu levels between clonally related murine OS cell lines differing in metastatic potential using atomic absorption (AA) spectrophotometry. Intracellular Cu levels were found to be significantly higher in low-metastatic K12 OS cells than in highly metastatic K7M2 (Figure 1(a)). Atomic absorption spectrophotometry was also performed on the FBS lot used for all experiments, DMEM, culture media (DMEM with 10% FBS), and culture media containing Dis (3 *μ*M), CuCl_2_ (1 *μ*M), and Dis + CuCl_2_ (0.1 + 0.25 *μ*M) (Figure 1(b)). Not surprisingly, the main Cu source was identified as FBS in our cell culture. Both K12 and K7M2 were treated with Dis, CuCl_2_, and Dis + CuCl_2_ over 24 hours. Intracellular Cu concentrations of K12 were significantly higher than those of K7M2 after treatment with 1 *μ*M CuCl_2_ and 100 nM Dis + 250 nM CuCl_2_ (Figure 1(c)). Dis + CuCl_2_-treated K12 cells displayed significantly increased intracellular Cu levels compared to all other K12 treated groups. Dis + CuCl_2_ treated K7M2 did not display significantly increased Cu levels compared to all other K7M2 treated groups (Figure 1(c)).

### 3.2. Significantly Altered Expression Levels in Copper Pumps and Chemoresistance Factors Are Observed in K12 and K7M2 Cells

**Figure 2:**
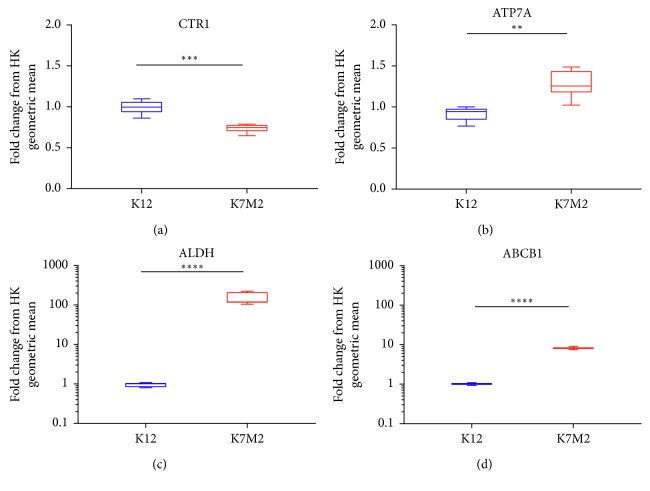
K7M2 displays altered expression of copper influx and efflux pumps as well as important chemoresistance factors compared to K12. K12 and K7M2 OS cell lines grown under identical culture conditions were processed for RNA extraction. cDNA generated was analyzed by qPCR for expression-level alterations between the OS cell lines and normalized to the geometric mean of housekeeper genes Nono, Rps17, and Rps18. (a) The Cu influx pump CTR1 was significantly downregulated in K7M2. (b) The Cu efflux pump ATP7A was significantly upregulated in K7M2 compared with K12. (c) Antioxidant enzyme and known cancer stem cell maker ALDH was significantly upregulated in K7M2 compared with K12 cells. (d) Additionally, the drug resistance multidrug pump ABCB1 was significantly upregulated in K7M2 compared with K12 cells. Molecular analysis was performed using technical triplicates from three separately cultured and processed samples (*n*=9). ^*∗∗*^*p* < 0.005; ^*∗∗∗*^*p* < 0.0005; ^*∗∗∗∗*^*p* < 0.0001.

Due to significant differences seen in endogenous intracellular Cu levels between K12 and K7M2, we next performed quantitative PCR to compare expression-level differences of genes important for Cu regulation and function in both OS cell lines. qPCR showed that highly metastatic K7M2 cells had significantly lower transcript levels of the Cu influx pump CTR1 compared with K12 (Figure 2(a)). Conversely, K7M2 showed significantly higher transcript levels of the copper efflux pump ATP7A compared with K12 (Figure 2(b)). Additionally, we tested transcript levels of important metastatic factors. In line with our previous findings [25], qPCR displayed significantly higher expression of aldehyde dehydrogenase (ALDH) in K7M2 compared to K12 (Figure 2(c)). Interestingly, K7M2 also displayed significantly higher expression of the ATP-dependent multidrug pump P-glycoprotein (ABCB1) compared with K12 (Figure 2(d)).

### 3.3. CuCl_2_ Potentiation of Disulfiram Results in Significant Selective Cytotoxicity to K12 Cells and Less to K7M2

**Figure 3:**
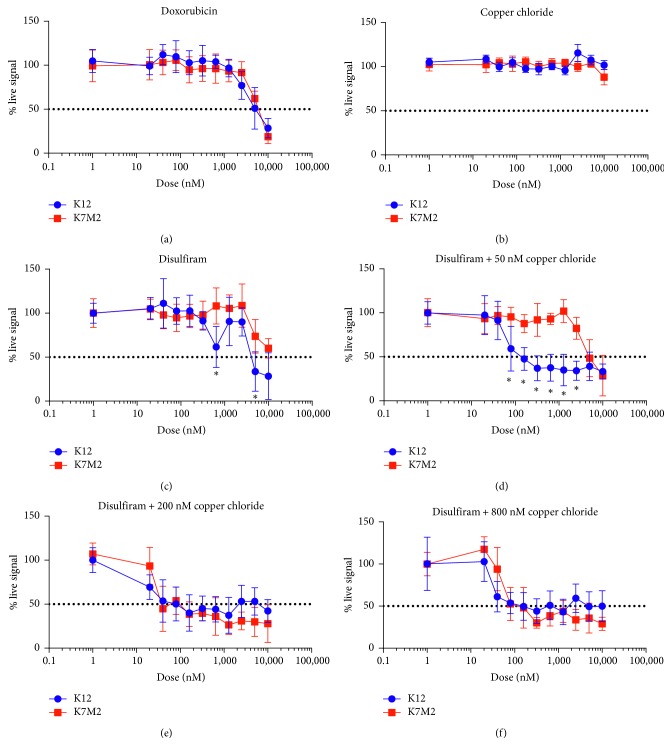
Copper potentiation of low-dose disulfiram selectively targets K12 cells compared to K7M2 cells. Both K12 and K7M2 cells were subjected to fold dilutions of Dox (a), CuCl_2_ (b), or Dis (c) single agent treatment over 24 hours. Additionally, cells were treated with fold dilutions of Dis + CuCl_2_ at fixed concentrations of 50 nM (d), 200 nM (e), or 800 nM (f). After treatment, cells were stained with LIVE/DEAD stain, and microscopy images were obtained. Live signal reduction from untreated controls was analyzed using FIJI image mean fluorescence measurements. 50 nM CuCl_2_ significantly decreased the live signal % in K12 than in K7M2 from 80 nM to 2.5 *μ*M dose of Dis. All cytotoxicity experiments were performed in technical triplicate with three separate cell cultures (*n*=9). ^*∗*^*p* < 0.05.

Given the significant and dramatic upregulation of ALDH expression in K7M2, as well as the retention of intracellular Cu levels in K12, we tested Cu-potentiated Dis cytotoxicity in both OS cell lines over 24 hours. The commonly administered anthracycline chemotherapy drug, Dox, showed equivalent cytotoxicity at high doses for K12 at IC_50_ 4.5 *μ*M and K7M2 at IC_50_ 5.2 *μ*M (Figure 3(a)). CuCl_2_ was not significantly cytotoxic in our LIVE/DEAD assays after 24 hours for the maximum dose (10 *μ*M) tested (Figure 3(b)). Interestingly, Dis as a single agent demonstrated significantly increased cytotoxicity against K12 compared with K7M2 at 640 nM. Dis treatment was also significantly more cytotoxic to K12 cells at the higher dose of 5 *μ*M (Figure 3(c)). Dis monotherapy did not reduce K7M2 viable cell signal beyond 50% from untreated controls up to 10 *μ*M. An addition of an 800 nM fixed CuCl_2_ supplement to Dis treatment resulted in clear potentiation in both K12 IC_50_ 60 nM and K7M2 IC_50_ 120 nM (Figure 3(f)). Further reduction to a 200 nM fixed CuCl_2_ supplement also resulted in clear enhanced cytotoxic effects of Dis in both cell lines with K12 IC_50_ 50 nM and K7M2 IC_50_ 60 nM (Figure 3(e)). Surprisingly, Dis supplemented with only 50 nM of CuCl_2_ resulted in clear potentiation and reduction in effective cytotoxic dose to K12 cells only, with significant differences in % live signal between OS cell lines from 80 nM to 2.5 *μ*M (Figure 3(d)). With this very limited dose of 50 nM CuCl_2_ supplement, the Dis IC_50_ was 160 nM for K12 and 4.4 *μ*M for K7M2, over a log difference in effective dose.

### 3.4. CuCl_2_-Potentiated Dis Requires Low-Dose Dox to Fully Eradicate K7M2 Cells *In Vitro*

**Figure 4:**
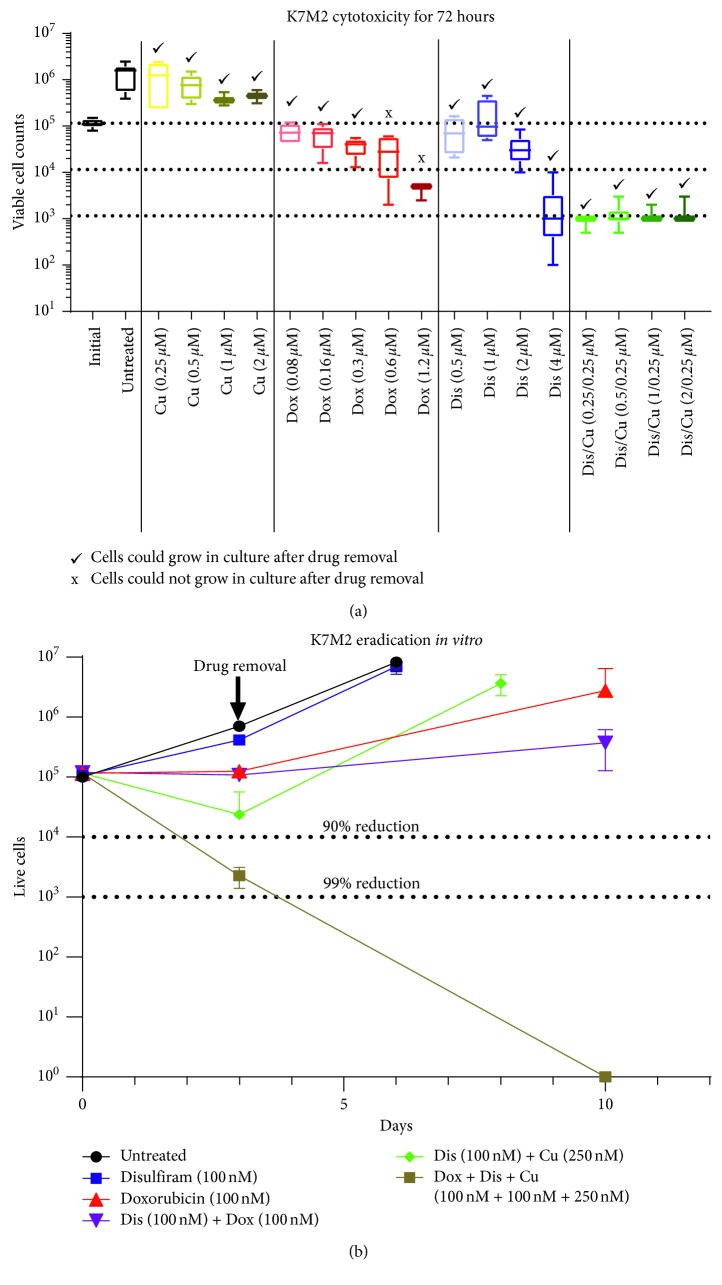
Combination treatment of doxorubicin, disulfiram, and copper chloride effectively reduces viability of K7M2 cells and eliminates recovery *in vitro*. (a) K7M2 OS cells were treated with CuCl_2_ (0.12–2 *μ*M), Dox (0.02–1.2, 5 *μ*M), Dis (0.5–4 *μ*M), and Dis + CuCl_2_ (0.12 and 0.25 *μ*M) for 72 hours. After treatment, trypan blue exclusion staining was performed to obtain viable cell counts. Cells were cultured again in fresh media without drugs present and monitored for cellular growth. Dis potentiated with CuCl_2_ clearly killed the most cells over 72 hours, but all could subsequently recover in culture. (b) Dox and Dis combination treatment also allowed for cellular recovery *in vitro*, but triple treatment with Dox, Dis, and CuCl_2_ resulted in over 90% killing over treatment duration and treated cells did not recover after drug removal. Experiments were performed using three independent cell cultures (*n*=3).

To assess the long-term treatment effects on cytotoxicity and cellular recovery potential of highly metastatic OS more accurately, K7M2 was treated over 72 hours with monotherapies and combination treatments of Dox, Dis, and CuCl_2_. After viable cell counts were obtained, cells were monitored for recovery in fresh, treatment-free, culture media. K7M2 did not display any cytotoxicity over 72 hours at any CuCl_2_ doses tested (0.12–2 *μ*M) (Figure 4(a)). High-dose Dis (4 *μ*M) reduced viable cell counts of K7M2 over 90% over 72 hours, but cells were able to grow in culture after drug removal. All CuCl_2_-potentiated Dis combination doses tested resulted in ∼99% reduction of viable K7M2 cells, but remaining populations were able to grow in culture after drug removal. K7M2 cells treated with 600 nM of doxorubicin displayed over 90% reduction in viable cells and were unable to grow in culture after drug removal (Figure 4(a)). We next treated K7M2 over 72 hours with sublethal doses of Dox (100 nM), Dis (100 nM), combination Dox and Dis (100 nM each), combination Dis + CuCl_2_ (100 nM and 250 nM respectively), and triple treatment of all three drugs. Triple treatment of Dox, Dis, and CuCl_2_ (100 nM, 100 nM, and 250 nM) resulted in ∼99% cell death after 72 hours of treatment, and, importantly, did not allow K7M2 cells to grow in culture after drug removal (Figure 4(b)).

## 4. Discussion

Here, we investigated endogenous Cu levels in murine OS cells with differing metastatic potentials and demonstrated that K12 cells had significantly greater endogenous intracellular Cu compared with the highly metastatic K7M2 cells. Evaluation of the expression levels for genes related to Cu metabolism and drug resistance mechanisms also demonstrated altered levels of cellular Cu transporters between the two cell lines, which is associated with retention of Cu in K12 and Cu export in K7M2. Finally, we evaluated the ability of CuCl_2_ to affect the cytotoxicity of Dis and Dox and found that combination treatment could produce a durable, cytotoxic effect on the highly metastatic K7M2 cell line.

OS prognoses have not improved in several decades despite multiple clinical trials, forcefully illustrating that our current Dox-based strategies have reached the limits of their efficacy. Novel strategies directed at deliberately chosen biological targets are needed to improve prognoses for these patients. The use of Dis could have many possible uses in improving treatment success in metastatic OS. As a monotherapy, Dis could aid in chelating systemic copper found in the blood of patients, thereby attenuating the Cu-associated metastatic mechanisms of OS. Dis could also be used as a chemosensitizer of metastatic OS cells, blocking ALDH antioxidant activity and improving the efficacy of ROS-inducing drugs like doxorubicin.

The ability of CuCl_2_ to augment the cytotoxic efficacy of Dis is well documented in the literature [30, 34–39]. There are multiple clinical trials in oncology using this combination [39–43], but not in sarcoma. We have demonstrated that Dis has the capacity to affect the viability and metastatic phenotype of metastatic sarcoma cells in several publications [38], but neither we nor others have combined CuCl_2_ with Dis in sarcoma cells until now. The difference in susceptibility to Dis is likely related to differences in Cu metabolism, although the exact mechanism is a source of ongoing research for us and others.

Dox-based neoadjuvant and adjuvant chemotherapy is accepted worldwide as the standard of care in OS treatment and is felt to be responsible for the improvement in OS survivorship compared with the prechemotherapeutic era [3, 14]. It is therefore unlikely that oncologists will embrace novel treatment schemes that do not include Dox. This is clearly illustrated by the most recent OS clinical trials, which were designed to compare the standard of care versus the standard of care plus a novel agent [3, 14]. With this reality in mind, we compared the ability of K7M2 cells to recover after treatment with Dox, Dis, CuCl_2_, or a combination of these. Only “triple treatment” with all three agents demonstrated cytotoxicity from which the cells did not recover. Interestingly, this effect was observed with low doses of Dox. This is clinically important because the cardiotoxic effects of Dox are known to be dose dependent. Any treatment strategy that decreases the cumulative dose of Dox would necessarily result in fewer cardiotoxicity complications.

There are several limitations to this study. First and most importantly, the experiments described above only used two murine OS cell lines for comparison. Although the K12 and K7M2 cell lines present a unique and powerful opportunity to compare related OS cells with differing metastatic phenotypes, it is still a small number of cell lines. We are presently seeking to rectify this deficiency by investigating these phenomena in established human OS cell populations, as well as primary cells from OS patients in our clinical practice with known metastatic histories.

## 5. Conclusions

In this study, we demonstrated differing levels of intracellular Cu in OS cell lines with different metastatic potentials. These differences are associated with the expression of genes related to Cu transport. Importantly, the addition of CuCl_2_ potentiates the cytotoxic effects of Dis. Finally, “triple treatment” with sublethal doses of Dox, Dis, and CuCl_2_ is the only experimental strategy we evaluated that leads to cell death from which cells could not recover. Based on these data, we theorize that highly metastatic and nonmetastatic OS cells utilize Cu differentially. We speculate that highly metastatic K7M2 cells actively pump Cu extracellularly to modulate the malignant processes of invasion and migration, possibly through matrix metalloproteinases (MMPs). Conversely, less metastatic K12 cells maintain higher amounts of intracellular Cu. This would suggest that low levels of intratumoral Cu and high levels of serum Cu would portend a more aggressive and metastatic phenotype. We plan to verify this in experimental samples and patient-derived serum and tumors. We also have observed a relationship between Dox, Dis, and CuCl_2_ that should be evaluated in our preclinical mouse model of metastatic OS. Future studies will serve to determine how these powerful interactions might be exploited to the benefit of patients with metastatic OS.

## Data Availability

The copper level, qPCR, and cell viability data used to support the findings of this study are available from the corresponding author upon request.
